# Graphene oxide as an additive to improve perovskite film crystallization and morphology for high-efficiency solar cells

**DOI:** 10.1039/c7ra12049d

**Published:** 2018-01-03

**Authors:** Xiaonan Zhang, Gengwu Ji, Dongbin Xiong, Zhenhuang Su, Bin Zhao, Kongchao Shen, Yingguo Yang, Xingyu Gao

**Affiliations:** Shanghai Synchrotron Radiation Facility, Shanghai Institute of Applied Physics, Chinese Academy of Sciences Shanghai 201204 China gaoxingyu@sinap.ac.cn +86-21-5955-3021 +86-21-3393-3199; University of Chinese Academy of Sciences Beijing 100049 China; Beijing Key Laboratory of Materials Utilization of Nonmetallic Minerals and Solid Wastes, National Laboratory of Mineral Materials, School of Materials Science and Technology, China University of Geosciences Beijing 100083 China; Department of Physics, Zhejiang University Hangzhou 310027 China

## Abstract

The quality of a perovskite film has a great impact on its light absorption and carrier transport, which is vital to improve high-efficiency perovskite solar cells (PSCs). Herein, it is demonstrated that graphene oxide (GO) can be used as an effective additive in the precursor solution for the preparation of high-quality solution-processed CH_3_NH_3_PbI_3_ (MAIPbI_3_) films. It is evidenced by scanning electron microscopy that the size of the grains inside these films not only increases but also becomes more uniform after the introduction of an optimized amount of 1 vol% GO. Moreover, 1 vol% GO also enhances the crystallization of perovskite film with intact preferential out-of-plane orientation as proven by 2-dimensional grazing-incidence X-ray diffraction. As a consequence of the improved film quality, enhanced charge extraction efficiency and optical absorption are demonstrated by photoluminescence (PL) spectroscopy and UV-visible absorption spectroscopy, respectively. Using 1 vol% GO, the fabricated champion heterojunction PSC with a structure of ITO/SnO_2_/perovskite/spiro-OMeTAD/Au shows a significant power conversion efficiency increase to 17.59% with reduced hysteresis from 16.10% for the champion device based on pristine perovskite. The present study thus proposes a simple approach to make use of GO as an effective and cheap addictive for high-performance PSCs with large-scale production capability.

## Abstract 2

The quality of perovskite film has a great impact on its light absorption and carrier transport, which is vital to improve high-efficiency perovskite solar cells (PSCs).

## Graphical abstract

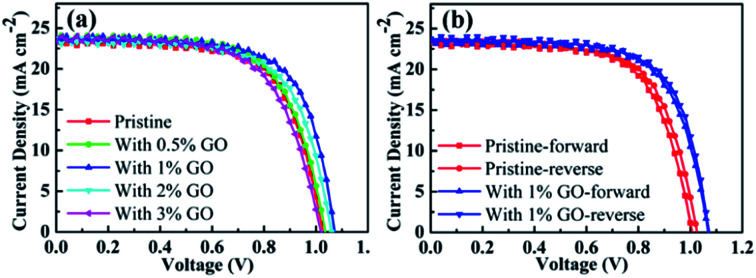

## Introduction

In the past few years, organic–metal halide perovskite solar cells (PSCs) have drawn massive attention as a new member of photovoltaic (PV) devices due to their excellent characteristics including broad light absorption range, ambipolar charge transport, and long carrier diffusion length.^1–4^ The power conversion efficiency (PCE) has skyrocketed from 3.8% to 22.1% since 2009.^5,6^ In addition, the performance can be further enhanced by meticulously optimizing perovskite composition, device structure, and the processing methods.^7–14^

Perovskite thin films can be prepared by one or two-step solution process, sequential deposition, and co-evaporation.^7–9,15^ A one-step solution process is a more preferable method than others owing to its simplicity and low annealing temperature. The perovskite crystallization process plays an important role in the formation of high quality one-step solution processed perovskite thin films for high performance PSCs. Poor crystallinity of a perovskite thin film is detrimental to its resultant PSC device performance due to a large amount of defects within the film leading to lots of serious recombination and voltage loss.^16–19^ Superior crystallinity of a perovskite thin film not only means a highly quality film with few defects, but also favors the interfacial contact between the perovskite thin film and the transport layer to enhance charge separation.^20,21^ Many factors are known to affect perovskite crystallization including the solvents used, solution concentration, precursor composition, post annealing temperature, and processing environment, which can be adjusted to improve crystallization for high quality perovskite thin films.^22–25^ Additives intentionally added into the precursor solution for the preparation of perovskite thin films are known to be able to effectively enhance the perovskite film crystallization and morphology for high performance PSCs.^26,27^ For instance, A. K. Y. Jen *et al.* introduced 1,8-diiodooctance (DIO) as an additive into the precursor solution during the film fabrication process, and DIO could temporarily coordinate with Pb^2+^ during crystal nucleation and growth, which tends to promote homogenous nucleation and likely modifies interfacial energy favorably.^17^ Similarly, Ding *et al.* adopted NH_4_Cl as an additive to fabricate perovskite thin films. The films turned out to be highly crystallized with excellent morphology, leading to remarkably enhanced device performance.^28^ Chen *et al.* found that 1-chloronaphthalene (CN) additive was beneficial to regulate the crystallization transformation kinetics of perovskite for high-quality perovskite thin films.^29^ Huang *et al.* introduced organic halide salts as a processing additive to modulate the morphology and crystallinity of the perovskite light-absorbing layer in perovskite/fullerene planar-heterojunction solar cells, which also serves as an interfacial modifier to improve the electrical contact of the [6,6]-phenyl-C_61_-butyric acid methyl ester (PCBM)/Al electron-collecting electrode.^30^ Su *et al.* used poly(ethyleneglycol) (PEG) additive to tune the morphology of the perovskite layer by retarding the growth and aggregation of perovskite crystals with the formation of a continuous and uniform film.^31^ Wu *et al.* improved the quality of the perovskite thin films by adding PCBM into PbI_2_ precursor solution for a low-temperature two-step solution process, and found that PCBM played a critical role in filling pinholes and vacancies, resulting in a film with large grains and fewer grain boundaries.^32^

On the other hand, graphene oxide (GO) has attracted a great deal of attentions due to its excellent electronic properties as well as its multi-functionalities, reliability, low production costs, large-scale production capability, and good dispersibility in many solvents. GO is a layered material with various oxygen-containing functional groups (carboxyl, carbonyl, phenol, lactone, and quinone) on its basal plane and edge. The distribution of hydrophilic and hydrophobic properties induced by its functional groups from the edge to the center make it an ideal surfactant.^33–36^ In addition, its heterogeneous chemical and electronic structures make GO a potential material for retarding electron recombination in high-performance PSCs. According to previous works, GO has already been demonstrated as an excellent interfacial material.^37–39^ For example, Wang *et al.* improved the photovoltaic performance of PSCs using GO as an amphiphilic modifier to enhance the interface contact between perovskite and the hole transport layer.^34^ The introduction of ammonia modified GO layer into PSC devices by Yang *et al.* improved their device performance and perovskite structure stability.^40^ Sun *et al.* employed GO as hole conductor on perovskite thin films in inverted planar heterojunction PSCs, which improved the perovskite crystallization with enhanced hole extraction.^41^ There are also reports about GO as a better hole transport layer than PEDOT:PSS to achieve enhanced PSC performance.^42,43^ In addition, Ding and Yuan *et al.* reported enhanced efficiency of mesoscopic perovskite solar cells by adding graphene quantum dots (GQDs) as additive into precursor to passivate the grain boundaries of CH_3_NH_3_PbI_3_.^44^ Nevertheless, the preparation of GQDs usually requires special equipment and suffers low production yield as well as critical synthesis conditions.^45–48^ Therefore, GQDs are unlikely a promising additive candidate for low-cost large area production of PSCs. To the contrary, GO itself can be easily and cheaply synthesized in large quantity, which could be a better additive candidate in the preparation of high quality perovskite thin films in PSCs.^35,36,49–51^ Indeed, Hagfeldt *et al.* introduced nitrogen-doped reduced graphene oxide (N-RGO) into the perovskite layer of PSCs, which tuned both morphological features and recombination dynamics, resulting in better PSC performance.^52^ Moreover, they found that native RGO barely increase the grain size and optical absorption but still enhance the device performance due to surface-passivation of the perovskite by graphene sheets resulting in improved hole selectivity and reduced recombination at the perovskite/spiro-OMeTAD interface. In this work, it is further proven that the crystallization of perovskite thin films prepared by one-step method can be improved simply by adding GO into their perovskite precursor solution. Using an optimization amount of 1 vol%, GO is demonstrated to promote the perovskite film quality with improved crystallization as well as large and uniform grains. Besides, it is also found that the incorporation of GO efficiently promote the charge extraction at perovskite/spiro-OMeTAD interfaces which may be due to the superior electronic properties of GO and the improved interfacial contact. Finally, the fabricated champion heterojunction PSC with a structure of ITO/SnO_2_/perovskite/spiro-OMeTAD/Au using 1 vol% GO shows significant power conversion efficiency increase from 16.10% for that based on pristine perovskite to 17.59%. The present study demonstrates GO as an effective and cheap addictive for high-performance PSCs with large-scale production capability.

## Results and discussion

### Photovoltaic performance

**Fig. 1:**
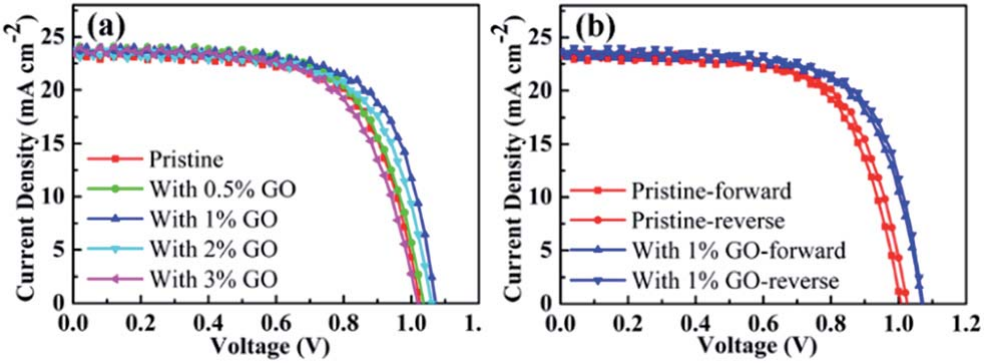
(a) *J*–*V* curves of the fabricated champion PSCs with different amount of GO in the perovskite precursor solution; (b) the forward and reverse scans for the champion pristine PSC without GO and that with 1 vol% of GO.

To find out the effects of GO on PSC performance, current–voltage (*J*–*V*) curves measured on the fabricated champion devices based on MAPbI_3_ using different amount of GO with a concentration up to 3 vol% are reported in Fig. 1. Their photovoltaic parameters derived from Fig. 1(a) are listed in Table 1. The pristine MAPbI_3_ champion device exhibits an open circuit voltage (*V*_OC_) of 1.026 V, a short circuit current density (*J*_SC_) of 23.41 mA cm^−2^, a fill factor (FF) of 67.08%, and a PCE of 16.10%, whereas the MAPbI_3_ champion device with 1 vol% of GO achieves a *V*_OC_ of 1.072 V, a *J*_SC_ of 23.73 mA cm^−2^, a FF of 69.14%, and a PCE of 17.59%, which obviously is the best among all the fabricated champion devices with an amount of GO from 0 to 3 vol%. It can be seen from Table 1 that *V*_OC_, FF, and PCE increase with the GO concentration internally and then drops quite fast after reaching their maxima at an amount of 1 vol%, and that *J*_SC_ increases similarly with the GO concentration but reaches its maximum at 2 vol%. In addition, Fig. 1(b) reports the reverse and forward scanned current–voltage curves for the pristine MAPbI_3_ PSC and that with 1 vol% of GO, which clearly indicates that the introduction of 1 vol% GO also reduces the hysteresis effectively. For a better comparison, their device parameters for both forward and reverse scans are summarized in Table 2. Therefore, all these results indicate that 1 vol% of GO is the most effective addictive concentration to enhance the PSCs performance with reduced hysteresis.

**Table 1:** Photovoltaic parameters of the ITO/SnO_2_/MAPbI_3_/spiro-OMeTAD/Au PSCs with different amount of GO

GO (vol%)	*J*_SC_ (mA cm^−2^)	*V*_OC_ (V)	FF (%)	PCE (%)	PCE_AVE_ (%)
0	23.41	1.026	67.08	16.10	14.68 ± 1.01
0.5	23.50	1.036	67.68	16.48	15.14 ± 0.72
1	23.73	1.072	69.14	17.59	16.35 ± 0.72
2	23.81	1.062	65.88	16.66	14.42 ± 1.06
3	23.44	1.019	65.31	15.59	13.36 ± 1.46

**Table 2:** The device parameters for the champion pristine PSC without GO and that with 1 vol% of GO derived from both forward and reverse scans in Fig. 1(b)

GO (vol%)	*J*_SC_ (mA cm^−2^)	*V*_OC_ (V)	FF (%)	PCE (%)
0-Forward	23.21	1.007	66.69	15.59
0-Reverse	23.41	1.026	67.08	16.10
1-Forward	23.45	1.069	68.50	17.17
1-Reverse	23.73	1.072	69.14	17.59

### SEM results

**Fig. 2:**
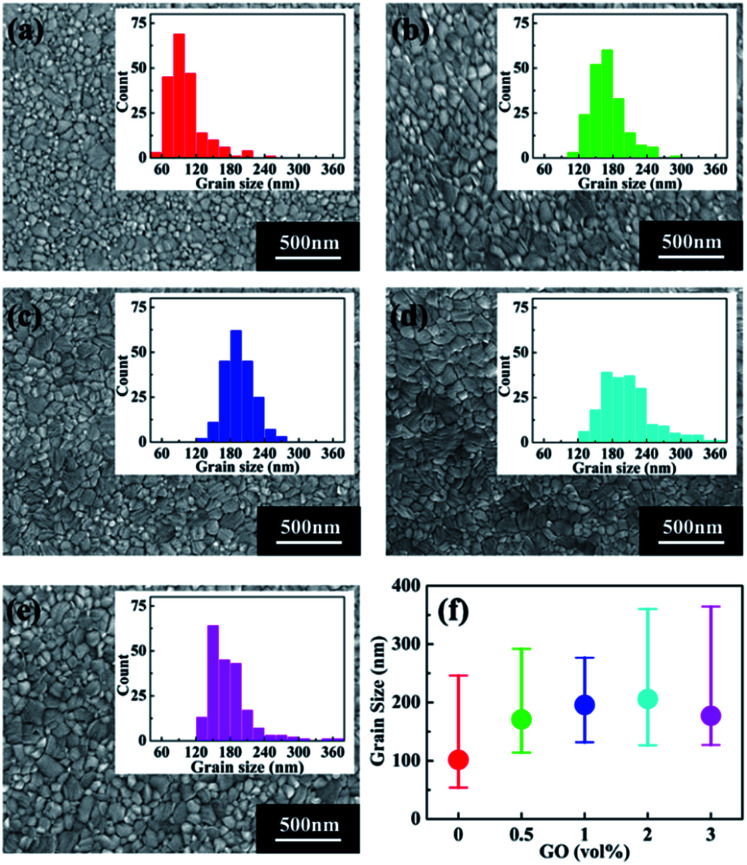
SEM images of perovskite thin films (a) pristine, with (b) 0.5 vol%, (c) 1 vol%, (d) 2 vol%, and (e) 3 vol% GO in the perovskite precursor. The insert in each image shows the corresponding grain size distribution. (f) The average grain size as the function of GO amount.

To study the possible reason for the PSC performance enhancement brought by GO, scanning electron microscopy (SEM) was conducted to investigate the morphology of perovskite thin films with different amount of GO, which are shown in Fig. 2(a)–(e). The insert in each SEM image is the grain size distribution of the corresponding film. For a better comparison, the average grain size as the function of GO amount is reported in Fig. 2(f). From these results, the average grain size of the pristine perovskite thin film is 100 nm, which quickly becomes larger with the increasing GO amount till 1 vol% to about 200 nm. After that, the average size still increases slightly at 2 vol% and drops at 3 vol%. It is also noticed that the film with 1 vol% GO has the narrowest grain size distribution indicating this film has the most uniform morphology among all the films.^53^

### GIXRD results

**Fig. 3:**
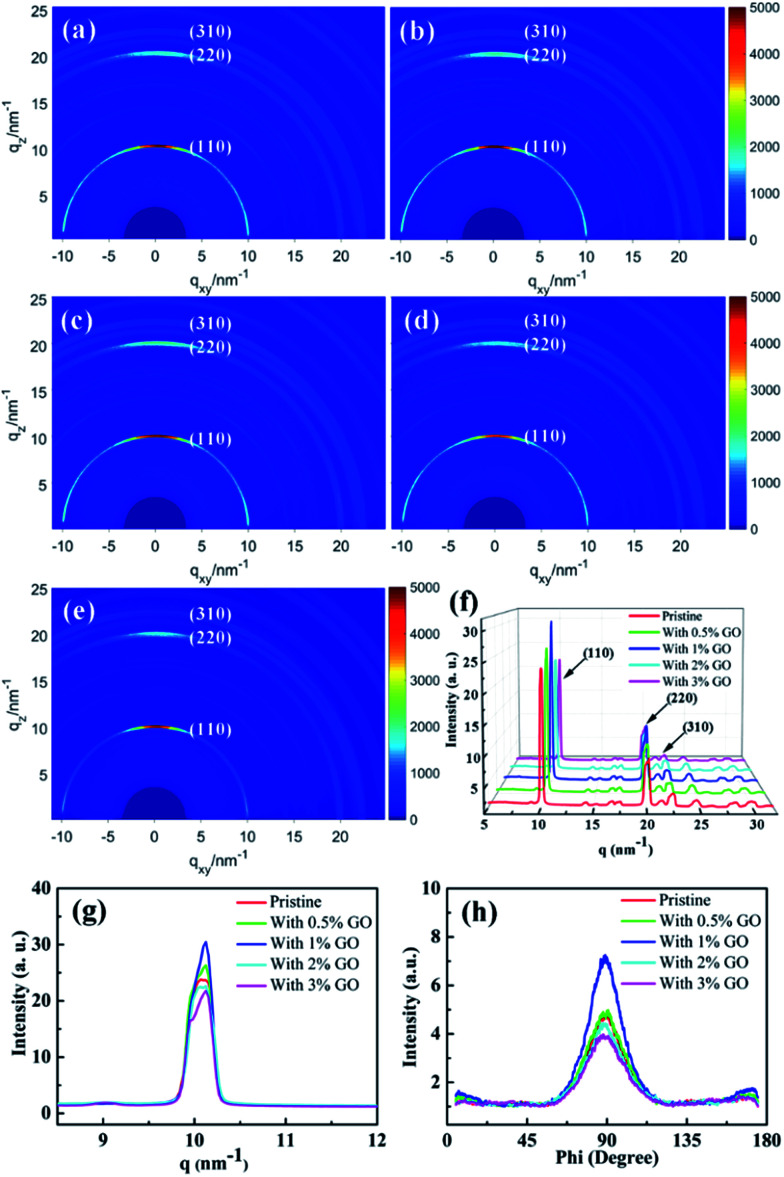
Two-dimensional GIXRD patterns of (a) the pristine perovskite thin film, (b) that with 0.5 vol%, (c) 1 vol%, (d) 2 vol%, and (e) 3 vol% GO in perovskite precursor, respectively; the azimuthally integrated intensity profiles derived from the diffraction patterns for the different films in (f) and these profiles around (110) peak in (g), respectively; (h) the radially integrated intensity profiles derived from the diffraction patterns at *q* ≈ 10 nm^−1^ with different amount of GO.

To investigate the crystallization of the fabricated perovskite film directly, two-dimensional GIXRD measurements were performed on the perovskite thin films with different amount of GO on the ITO/SnO_2_ substrates. Fig. 3(a)–(e) report the obtained GIXRD patterns, which show scattering rings at *q* ≈ 10, 20, and 23 nm^−1^, consistent with the typical (110), (220), and (310) tetragonal MAPbI_3_ diffraction, respectively.^54^Fig. 3(f) reports the azimuthally integrated intensity profiles derived from the diffraction patterns for the different films, which indicates quantitatively their crystallization degree. The dominant (110), (220), and (310) peaks in Fig. 3(f) demonstrate that these films possess good crystallization degree with large grains, which are proven more clearly by the strong and sharp (110) peak in Fig. 3(g). It is clear that the film with 1 vol% GO displays the highest and sharpest perovskite (110) diffraction peak due to its highest crystallization degree, which is obviously vital to the enhanced performances of the present optimized PSCs.^41,54,55^ To study the crystallization preferential orientation, the radially integrated intensity profiles derived from the diffraction patterns at *q* ≈ 10 nm^−1^ for the perovskite (110) plane in the films with different amount of GO are present in Fig. 3(h). The dominant peaks at an azimuth angle of 90° demonstrate that all the films exhibit a preferential out of plane orientation, which favor the interfacial charge transporting.^56–58^ Thus, the presence of GO did not change the out-of-plane preferential order of the perovskite crystallization. It becomes clear that 1 vol% is the optimized amount of GO to achieve the best quality perovskite film among all these films with best crystallization and morphology as evidenced by the SEM and GIXRD results. The excellent electronic properties of GO and/or the functional groups on the GO surface make GO likely serve as efficient chemical reaction and crystallization nucleation centers during the perovskite film growth, promoting chemical reaction and enhancing crystallinity with large grains and few defects. When the amount of GO is less than 1 vol% and they should be able to distribute uniformly in the precursor solution, the increasing amount of GO means more reaction and nucleation centers during the formation of perovskite to facilitate the film crystallization leading to larger grains and more uniform morphology. In fact, it was noticed that the prepared precursor solution with less than 3 vol% GO stayed clear and transparent for several days, which proves that GO can distribute uniformly in the precursor solution. With more than 1 vol% GO present in the precursor solution, however, GO could segregate occasionally during the formation of perovskite leading to randomly distributed and even fewer reaction and nucleation centers causing worse crystallization and morphology. While the optimal amount of 1 vol% GO is proven to achieve most enhanced crystallization and best morphology in the perovskite films, it will be shown later to facilitate carrier extraction and reduce exciton recombination as well as improve the optical absorption. All these achievements induced by 1 vol% GO should be the main reason leading to the best PSC device.

### UV-vis and EQE spectra

**Fig. 4:**
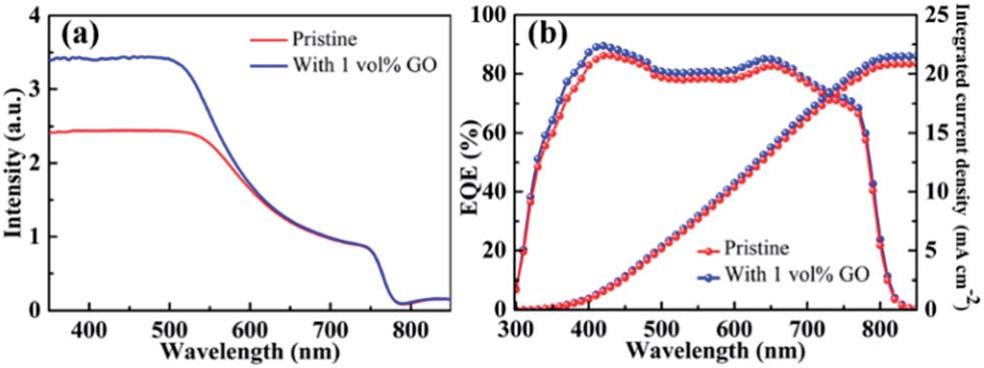
(a) UV-vis absorption spectra of a pristine perovskite film and another with 1 vol% GO; (b) EQE spectra with integrated current density of the PSC based on the pristine perovskite film and that with 1 vol% GO.

Fig. 4(a) reports the UV-vis absorption spectrum of a pristine CH_3_NH_3_PbI_3_ film directly spin-coated on clean glass and that of another film with 1 vol% GO. The obvious absorption decrease after 500 nm as well as the absorption onset at about 790 nm in these two spectra are characteristics of MAPbI_3_ spectrum.^59^ In comparison with the pristine film, the optical absorption for the film containing GO is almost identical to that for the pristine film from 600 to 800 nm but higher in the near UV region from 350 to 550 nm, which should be mainly due to the improved crystallinity of perovskite thin film by GO as evidenced by the GIXRD and SEM leading to higher *J*_SC_ in the PSC containing GO. Fig. 4(b) reports the EQE spectra with integrated current density of PSCs based on a pristine perovskite film and another with 1 vol% GO fabricated following the same processes described before. The broad spectral response in the range of 350–800 nm agrees with the absorption spectra of perovskite thin films in Fig. 4(a). The integrated *J*_SC_ values are calculated to be 20.84 mA cm^−2^ for the PSC based on the pristine film and 21.49 mA cm^−2^ for that with 1 vol% GO, respectively, which proves again that GO is an efficient addictive to improve the PSC performance as demonstrated in *J*–*V* measurements.

### PL and TRPL

**Fig. 5:**
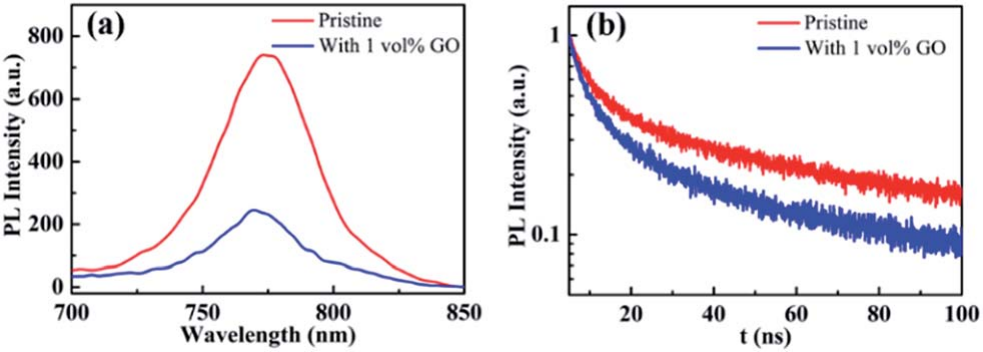
(a) Steady-state photoluminescence (PL) and (b) time-resolved photoluminescence (TRPL) spectra of glass/MAPbI_3_/spiro-OMeTAD without or with 1 vol% GO.

Photoluminescence characterization was widely used in photovoltaic related fields to obtain information about charge carrier dynamics in the active layer or at the interface between the active layer and transport layers. In order to investigate the hole extraction efficiency from perovskite to the transport layer, perovskite films deposited on glass substrate and then coated with a spiro-OMeTAD hole transport layer were used for the steady-state photoluminescence (PL) and time-resolved photoluminescence (TRPL) measurements. Before the PL experiments, UPS spectra of these films were measured, which shows that the work function, the valance band maximum, and other valence band photoemission features of the perovskite films are not changed by the introduction of GO, indicating a few vol% GO addictive barely changes the electronic structures of the perovskite films. From the normalized steady-state PL spectra in Fig. 5(a), it is clear that the PL intensity of the perovskite film with 1 vol% GO is much lower (*i.e.*, high PL quenching) than that of the pristine film, which is associated with the efficient charge separation and extraction (or injection) from the photoactive perovskite to the charge-transport layer. Therefore, Fig. 5(a) indicated that GO can obviously improve the charge extraction, which may be related to the superior electronic properties of GO or improved interface contact between high quality perovskite and hole transport layer. As can be seen from the TRPL spectra in Fig. 5(b), the film with GO decay much faster than the pristine film. To quantitatively analysing the carrier lifetime, the TRPL profiles can be fitted by the following bi-exponential function:
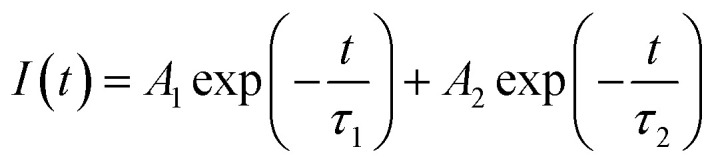


In the function, *τ*_1_ and *τ*_2_ represent decay characteristic time for two decay components: the fast decay *τ*_1_ reflects diffusion of initially photo-generated excitons into defects or charge selection layers, and the slow decay *τ*_2_ is associated with the exciton lifetime of perovskite thin film. General, a small *τ*_2_ value will indicate a fast exciton- or hole-diffusion process.^60–63^ The fitted parameters derived from the two spectra are summarized in Table 3. As shown in Table 3, both *τ*_1_ and *τ*_2_ for the film with GO are smaller than the corresponding values for the pristine film, proving that GO obviously promote hole extraction and transport. The enhanced carrier extraction must be associated with the GO in the perovskite thin film. First of all, the better film morphology could improve the contact between perovskite and transport layer spiro-OMeTAD, which promotes the hole injection into spiro-OMeTAD, leading to reduce recombination at the interface.^56^ Secondly, the large and uniform grains will reduce grain boundary and defects, which allow the exciton transport more easily. Thirdly, GO possibly reside at the grain boundaries or surface could be effectively bonded with MAPbI_3_ and passivate the dangling bonds to eliminate electron raps, which will facilitate the charge extraction. In addition, the electrons will transfer faster through GO in the perovskite film to separate more quickly from the holes, which could explain the increases of *J*_SC_, *V*_OC_ and FF. As exciton could transport in the film more freely leading to a longer diffusion length as well as faster a diffusion rate, hysteresis is also eliminated effectively as demonstrated before.

**Table 3:** Fitted parameters derived from TRPL spectra

GO (vol%)	*τ*_1_ (ns)	*τ*_2_ (ns)
0	4.07	34.95
1	3.86	26.92

## Experimental

### PSCs fabrication

To prepare perovskite precursor solution, 269 mg methylammonium iodide (MAI, 99.9%, MaterWin New Materials Co. Ltd, Shanghai) and 780 mg lead(ii) iodide (PbI_2_, 99%, Aldrich) powder were dissolved in 880 μL *N*,*N*-dimethylformamide (DMF, anhydrous, 99.8%, Sigma-Aldrich) solution and then added by 120 μL dimethyl sulfoxide (DMSO, anhydrous, 99.9%, Sigma-Aldrich). The precursor solution was stirred for 1 h at 60 °C before adding GO. GO was fabricated by a modified Hummers method as reported.^64^ The as-prepared GO was ground into fine powder, which was dispersed in DMF at 0.5 mg mL^−1^ and ultrasonicated for 4 hours. Finally, different amount of GO solution was added into precursor solution to prepare a mixed solution with different GO concentration (0.5, 1, 2, and 3 vol%), which was stirred at 60 °C for another 2 h before usage.

Indium tin oxide (ITO) substrates (12 Ω sq^−1^, 1.5 cm × 1.5 cm) were cleaned sequentially in an ultrasonic solvent bath of detergent, deionized water, acetone, and alcohol. After dried in a stream of nitrogen, the substrates were treated in Ultraviolet Ozone (UVO) cleaner for 10 min. Then, the 2.67% SnO_2_ solution was coated on ITO, and annealed at 150 °C for 30 min in air. The substrates were then transferred into a glove box (H_2_O and O_2_ < 1.0 ppm) filled with N_2_. Perovskite layer was formed by spin-coating precursor solution at 3000 rpm for 30 s, 1 mL of ether was dripped just after the spin coating started. Then the films were annealed at 100 °C for 5 min. After cooling to room temperature, 2,2′,7,7′-tetrakis(*N*,*N*-bis(*p*-methoxyphenyl)amino)-9,9′-spirobifluorene (spiro-OMeTAD) solution doped with bis(trifluoromethylsulfonyl)-imide lithium salt (Li-TFSI) and *tert*-butylpyridine (TBP) was spin-coated at 2000 rpm for 45 s. In the end, the films were transferred into a vacuum chamber, where gold electrodes with a thickness of 100 nm were evaporated under high vacuum (<5 × 10^−6^ Torr) through a shadow mask. Therefore, the fabricated planar PSC devices have an architecture of ITO/SnO_2_/perovskite/spiro-OMeTAD/Au.

### Characterization

Synchrotron based GIXRD (grazing-incidence X-ray diffraction) was performed at the BL14B1 beamline endstation of Shanghai Synchrotron Radiation Facility (SSRF), using X-ray with a wavelength of 0.06887 nm with an incident angle of 0.20°.^65^ The diffractive patterns were recorded by an area detector (charge coupled device, CCD). A cold field-emission scanning electron microscope (SEM, Hitachi S-4800) was used to obtain top view morphology. UV-vis spectra was conducted by a UV-VIS-NIR 3600 spectrophotometer (Shimadzu). While a xenon light source solar simulator (450 W, Oriel, model 9119) with an AM 1.5G filter (Oriel, model 91192) was used to give an irradiance of 100 mW cm^−2^ at the surface of the solar cells, their photovoltaic characterization was carried out by using a Keithley 2400 digital source meter. The effective area of each cell was 0.102 cm^2^ defined by masks for all the photovoltaic devices. External quantum efficiencies (EQE) were measured by an Enli Technology (Taiwan) EQE measurement system. Ultraviolet photoemission spectroscopy (UPS) was performed in an ultrahigh vacuum (UHV) system by using a PHOIBOS 100 analyzer together with a helium light lamp (He I: 21.2 eV). Transient-state photoluminescence (PL) was measured by FLS980 (Edinburgh Instruments Ltd.) with an excitation at a wavelength of 470 nm.

## Conclusions

In conclusion, by adding 1 vol% GO into the perovskite precursor solution to prepare the perovskite films, performance of resultant PSCs can be enhanced with a PCE increase from 16.10% to 17.59% with significantly reduced hysteresis effects. These performance achievements can be attributed to the crystallization and morphology improvement brought by GO as additive, which not only facilitates carrier extraction and reduce exciton recombination but also improves the optical absorption. Thus, GO is proven to be an effective additive for the preparation of high quality solution-processed perovskite towards high-efficiency PSC for large-scale production capability.

## Conflicts of interest

There are no conflicts to declare.

## Supplementary Material
